# Direct and Second Hand Cigarette Smoke Exposure and Development of Childhood Asthma

**DOI:** 10.15436/2378-6841.16.1122

**Published:** 2016-12-17

**Authors:** Srirupa Hari Gopal, Shyamali Mukherjee, Salil K. Das

**Affiliations:** 1 Department of Biochemistry and Cancer Biology, Meharry Medical College, Nashville, TN, USA; 2 Department of Professional Education, Neurosciences & Pharmacology, Meharry Medical College, Nashville, TN, USA

**Keywords:** Direct and second hand tobacco smoke, Pregnancy, Childhood asthma

## Abstract

This is a comprehensive review about the role of direct and second hand cigarette smoke exposure in the development of childhood asthma. Smoking, both during pregnancy and postnatal have an adverse impact on the infant’s chances of developing respiratory illness. Second hand smoke exposure has also known to cause worsening of childhood asthma with an impact on hospital admissions. Correlation between maternal second hand smoke exposure during pregnancy and development of childhood asthma has also been investigated. It is, thus essential to address this prenatally as well as post-natal by reducing smoking as well as smoke exposure.

## Introduction

Tobacco smoke contains more than 7,000 chemicals, including hundreds that are toxic and about 70 that can cause cancer (CDCP, 2014). Smoking accounts for the loss of 57 million disability-adjusted life-years (DALYs), becoming one of the top 10 risk factors for mortality (Global Health Risks-WHO, 2009). Health expectancy based on long standing illness and quality of life is reduced for smokers when compared with never smokers, with a significant effect on health care needs. Besides the detrimental effects of tobacco smoking, second hand smoke exposure over a period of time also has significant adverse-effects on health. Second hand smoke arises from burning tobacco products, such as cigarettes, cigars, or pipes (CDCP, 2014). It is also smoke exhaled by the person smoking (NAS, 2010; NTP, 2014). Cotinine is produced when the body breaks down the nicotine found in tobacco smoke and second hand smoke can be detected by measuring its levels in saliva, blood or urine. During 2011–2012, about 58 million nonsmokers in the United States were exposed to secondhand smoke (Homa, et al., 2015). Since 1964, approximately 2,500,000 nonsmokers have died from health problems caused by exposure to secondhand smoke. There is no risk-free level of second hand smoke exposure; even brief exposure can be harmful to health (CDCP, 2010; RSG-CDCP, 2010; CDCP, 2014).

Infants and toddlers face substantial consequences of both maternal smoking and second hand smoke exposure, due to their developing respiratory and immune system. It is known that exposure to environmental tobacco smoke among children, especially due to maternal smoking, may be a significant risk factor for the development of childhood asthma (Das, 2003). During 2011–2012, 2 out of every 5 children ages 3 to 11 in the United States—including 7 out of every 10 black children were exposed to secondhand smoke regularly (Homa, et al., 2015). The fetal lung, during its developing stage, is unable to protect itself against inhaled foreign substances as efficiently as an adult’s which in turn may reduce the ability of the respiratory and other systems to protect themselves against other environmental insults, leading to an increase in the potential for disease.

### Pathogenesis of smoke Exposure

The developing fetus is largely dependent on the mother for protection against oxidants. Because cigarette smoke is high in oxidants, it drastically depletes the mother’s plasma and tissue antioxidants (Fraga, et al., 1996). Furthermore, nicotine exposure results in a decrease in the activity of antioxidants such as superoxide dismutase (SOD), catalase, and vitamins C and E (Zaken, 2001). This imbalance of oxidants and antioxidants due to maternal smoke exposure is one of the most important causes of respiratory pathology in childhood illness. If not addressed earlier, it is maintained long after nicotine withdrawal and becomes worse with age (Bruin, et al., 2008).

Fetal exposure to nicotine has been shown to have many effects on lung structures and lung metabolism (Maritz, et al., 2011). In particular, prenatal tobacco exposure is known to alter airway structure: in a study of 32 infants that died of sudden infant death syndrome (SIDS), the distance between alveolar attachments in the intraparenchymal airways was larger in children whose mothers smoked during pregnancy than in the 8 children whose mothers did not smoke, which suggests that airways may have been narrower in the children exposed to maternal smoking, (Elliot, et al., 2003). Prenatal airway growth is a key predictor of adult lung function. It was investigated that a poor airway function measured shortly after birth in 123 infants predicted airflow obstruction in early adulthood (Stern, et al., 2007). The impairment of prenatal airway growth may be related to exposure to maternal cigarette smoke (CS) in utero.

In a recent mouse model to investigate a synergistic effect of exposure to Cigarette Smoke (CS) in utero and during adolescence on lung function by Drummond et al, it was found that in utero exposure to CS induced significant lung function impairment. These mice had a significantly lower compliance (p = 0.01), and a significantly higher elastance (p < 0.01) of the whole respiratory system than control mice (Drummond, et al., 2016).

In addition, oxidative stress also induces fetal programming which contributes to the development of adult-onset diseases (Luo, et al., 2006) Endothelial cells of blood vessels are damaged as early as during the first month of life of children exposed to second hand smoke and these effects can be detected during the first decade of life (Das, 2003). In a population representative study to investigate the association between prenatal tobacco exposure and telomere length in children (aged under 15 years with prenatal tobacco exposure), it was found that the telomere length in children with prenatal tobacco exposure was significantly shorter than in those with no exposure (mean T/S ratio = 24.9 [SD = 8.58] in exposed vs. 28.97 [14.15] in control groups; (P = 0.02) thus, leading on to premature aging and increased health risks (Ip, et al., 2016)

Thus, by this imbalance between oxidants and anti- oxidants, protection afforded to the developing fetus and fetal respiratory system by the mother is compromised, causing vulnerability to oxidative DNA damage and altering the development of the respiratory system.

### Maternal Smoking and its effects

Smoking during pregnancy accounts for a sizable number of infant deaths in the United States (Salibu, et al., 2003). It has been long known that maternal smoking causes increased risk of adverse outcomes in the infant. Smoking during pregnancy increases the neonatal health care costs (Adams, et al., 2002). Maternal cigarette smoking during pregnancy increases the risk of having a child with congenital digital anomaly (Man. et al., 2006). Children with tobacco smoke exposure have also an increased risk of low birth weight, Sudden Infant Death Syndrome (SIDS), asthma, bronchitis, pneumonia, middle ear infection, and other diseases (RSG, 2006).

Hollams et al. stated that maternal smoking during pregnancy resulted in increased risk of asthma and wheezing at age 14. This increased risk was not due to increased atopic sensitization or reduced lung function at this age. Exposure to maternal CS has been negatively associated with lung function in adolescents and young adults (Hollams, et al., 2014) and in early adulthood (Hayatbakhsh, et al., 2009). In a recent prospective cohort study conducted by Tanaka et al, 1354 Japanese mother-child pair study subjects were selected to investigate the association between prenatal smoking exposure and postnatal living with household smokers and the risk of atopic eczema (as defined according to the criteria of the International Study of Asthma and Allergies in Childhood (ISAAC) in Japanese children aged 23 to 29 months. It was found that prenatal smoking exposure was associated with an increased risk of physician-diagnosed atopic eczema (adjusted odds ratio (aOD) = 7.11, 95% confidence interval: 1.43 to 27.8), thus suggesting that maternal smoking during pregnancy may increase the risk of atopic eczema in young children (Tanaka, et al., 2016).

Previous studies have suggested that maternal smoking and personal smoking may have a synergistic effect on lung function decline, but were unable to separate the influences of prenatal and postnatal exposure to parental smoking (Upton, et al., 2004; Guerra, et al., 2013). A recent clinical study (Jaakkola, 2004) also revealed that maternal smoking in pregnancy increases the risk of asthma during the first 7 years of life, and only a small fraction of the effects seemed to be mediated through fetal growth. However, the risk of respiratory illness and death has been reported to be increased in infants of low birth weight for gestational age as well as diminished airway function. Small-for-gestation infants shortly after birth appears to be primarily mediated through impaired somatic growth (Lum, et al., 2001). On the other hand, maternal smoking has been correlated with small for gestational age fetuses and subsequent neuron-development (Soothill, et al., 1995). Thus, it is not clear if the perturbed lung development and greater incidence in asthma in offspring are simply due to the pleiotropic consequences of preterm birth, inflammatory and related changes in the lung, or if smoking is the cause of both low birth weight and of altered lung development. It is also interesting that maternal smoking affects fetal growth more in the male than the female fetus. A greater intrauterine growth velocity and a different hormonal milieu are postulated as possible explanations of the greater male susceptibility (Zaren, et al., 2000).

The effect of maternal smoking does not limit itself to just the gestational period but also has postnatal impact. Tobacco smoking and exposure to second-hand smoke are widely accepted to be causative factors for childhood asthma and chronic obstructive pulmonary disease (COPD) affecting nearly 3 billion people worldwide, predominantly in China, India, and Africa (COPD, 2007). Baheiraei et al. conducted a prospective cohort study on 51 cigarette smoke-exposed infants (exposed group) and 51 non-exposed infants (non-exposed group) and they were evaluated for weight, height and head circumference. Exposure to secondhand smoke was assessed through questionnaires and urinary cotinine levels. It was observed that based on urinary cotinine levels, the exposed group had lower body weight than the non-exposed group at two months (5258.82 ± 233.6 vs. 5592.1 ± 216.4 g, P < 0.001). Median height of infants in both groups were similar at baseline, but the non-exposed infants were taller than the exposed at four months after birth (p < 0.001). Furthermore, height growth of the non-exposed infants was more than another group from 3 – 5 days to two months after birth (P = 0.04) and also from 3 – 5 days to four months of age (P = 0.008) (Baheiraei, et al., 2015).

Environmental tobacco smoke, a major air pollutant, affects asthmatic children by impairing pulmonary function (Buczko, et al., 1984; Murray, 1986; Evans, et al., 1986; Weitzman, et al., 1990; Meijer, et al., 1996), enhancing airway reactivity (Buczko, et al., 1984; Murray, 1986; Menton, et al., 1986; Murray, 1989; Weitzman, et al., 1990; Pitman, 1992; Frischer, et al., 1992; Chapman, 1996) and increasing pulmonary morbidity (Chilmonczyk, et al., 1993). There is a significant association between parents who smoke and the urinary cotinine levels of their children (Willers, et al., 1992). Burke et al. (2012) conducted a systematic review and meta-analysis to provide estimates of the prospective effect of smoking by parents or household members during prenatal or postnatal periods, on the risk of wheeze and asthma at different stages of childhood. Based on a review of 79 prospective studies, It was found that exposure to pre- or postnatal passive smoke exposure was associated with a 30% to 70% increased risk of incident wheezing (strongest effect from postnatal maternal smoking on wheeze in children aged # 2 years, Odds Ratio (OR) = 1.70, 95% CI = 1.24–2.35, 4 studies) and a 21% to 85% increase in incident asthma (strongest effect from prenatal maternal smoking on asthma in children aged # 2 years, Odds Ratio (OR) = 1.85, 95% CI = 1.35–2.53, 5 studies). Household passive smoke exposure increased the risk of wheeze in children aged 2 years (Odds Ratio (OR) =1.35, 95% CI = 1.10–1.64, I2 = 64.5%, 9 studies) (Burke, et al., 2012).

Increased postnatal smoke exposure can also have significant impact on the infant’s respiratory status. Studies by Nyunoya et al. (2006) using normal human diploid lung fibroblasts showed, for example, that a single exposure to cigarette smoke inhibits normal fibroblast proliferation which is essential for lung repair and maintenance. Furthermore, multiple exposures to cigarette smoke induce irreversible senescence of these cells and consequently slower proliferation and thus damaged repair mechanisms (Nyunoya, et al., 2006). Normally, the respiratory epithelium lines the airways system and forms continuous barrier to the diffuse of inhaled airborne particles. Airway hyper reactivity may develop due to changes in allergen loads such as occupational allergens or mite associated allergens in the indoor environment (Hendrick, 1986; Fleming, 1987; Charpin, et al., 1988; Platts-Mills, 1989; Sears, et al., 1989) such hyper reactivity is exacerbated by air pollution and tobacco smoking (Andrae, et al., 1988; Tager, 1988). Maternal and paternal smoking contributes to changes in the external and in utero environments and therefore affects lung development during phases of high tissue plasticity. This may severely increase the vulnerability of the offspring to disease in adulthood (Gluckman, et al., 2010)

### Maternal Secondary smoke Exposure and its effects

Various adverse effects have been associated with both maternal smoking and postnatal secondary smoke exposure on child health, prompting inquiry into the effect of second hand smoke exposure during pregnancy. Previous studies have shown that infants born to nonsmoking women exposed to second hand smoke during pregnancy had lower birth weight compared to the non-exposed group. In a recent hospital based study conducted in Japan by Inoue et al. (2016) both parents’ smoking showed clear associations with LBW (odds ratio [OR] = 1.64, 95% confidence interval [CI] 1.18 – 2.27) and short birth length (−1 standard deviation [SD] OR = 1.38, 95% CI 1.07 – 1.79; −2 SD OR = 2.75, 95% CI 1.84 – 4.10) (Inoue, et al., 2016). Pregnant women who are exposed to secondhand smoke are estimated to be 23% more likely to experience still birth and 13% more likely give birth to a child with a congenital malformation. (Leonardi, et al., 2011).

In the aforementioned review by Burke et al. (2012) it was observed that prenatal second hand smoke exposure was associated with a 52% increased risk of wheeze in children aged 5 to 18 years (Odds Ratio (OR) = 1.52, 95% CI = 1.23–1.87, I2 = 21.1%, 5 studies) (Burke, et al., 2012). In a study conducted recently by Harju et al. (2016) it was found that asthma prevalence was highest among families where either the mother or father smoked (7.8 % and 8.1 %). The risk of asthma was highest among children when both parents smoked (Adjusted Odds Ratio (AOR) 3.7, 95 % Cl 3.2 – 4.4) compared with children of non-smoking parents. The risk of asthma was also significantly increased in families when either one of the parents smoked (mother: AOR 1.7, 95 % Cl 1.2 – 2.2, father: AOR 2.9, 95 % Cl 2.5 – 3.3) compared with children of nonsmoking parents. Among families where the father smoked and the mother quit during pregnancy, the risk of asthma among children was still significantly increased (AOR 2.8, 95 % Cl 2.3 – 3.4). Thus, it was observed that paternal smoking represented an even more significant risk of asthma among offspring than maternal smoking (Harju, et al., 2016). Further, there is increasing evidence that prenatal exposure has a greater impact on pediatric asthma than postnatal exposure (Kumar, 2008). However, the precise effect of pre- and postnatal exposure to ETS on childhood asthma and other adverse outcomes are difficult to identify, since it is likely that women who smoke during pregnancy continue smoking after delivery (Tong, et al., 2009).

A study conducted by Simons, et al. (2014) determined a longitudinal association between maternal second hand exposure during pregnancy and impact on the development of childhood asthma. It was found that during pregnancy 5% of the mothers smoked and 6.2% were nonsmokers, but were exposed to second hand smoke. When adjusting for the factors such as male gender, preterm birth, maternal asthma, Breastfeeding, maternal smoking or second hand smoke exposure during pregnancy was associated with a 30% increase in adjusted hazard of childhood asthma development. Another recent study associated fetal exposure to maternal passive smoking on current wheezing although the effect was only observed in those exposed in the third trimester of pregnancy (Paraskevi. et al., 2009). Further, Lee et al. (2012) showed significant association of fetal exposure to maternal second hand smoke with wheezing, allergic rhinitis, and eczema (Lee, et al., 2012).

## Conclusion

Tobacco smoke in any form is detrimental for normal growth and development of an infant. Since smoking during and after pregnancy has demonstrated effects on disease progression in infants, mothers have to be counseled appropriately in order to prevent respiratory pathology in their off springs. Since second hand smoke has a role to play in this disease process, efforts to counsel the whole family against smoking to prevent diseases like asthma must be undertaken. This can prevent the disease process from developing, as well as control progression, if initial insult has already occurred. Not smoking during pregnancy or quitting smoking is critical to improving the health outcome of our future generations.
